# Phytochemicals Content, Antioxidant Activity and Acetylcholinesterase Inhibition Properties of Indigenous *Garcinia parvifolia* Fruit

**DOI:** 10.1155/2013/138950

**Published:** 2013-10-31

**Authors:** Siti Hawa Ali Hassan, Jeffrey R. Fry, Mohd Fadzelly Abu Bakar

**Affiliations:** ^1^Institute for Tropical Biology and Conservation, Universiti Malaysia Sabah, Jalan UMS, 88400 Kota Kinabalu, Sabah, Malaysia; ^2^School of Biomedical Sciences, University of Nottingham Medical School, Queen's Medical Centre, Nottingham NG7 2UH, UK

## Abstract

*Garcinia parvifolia* belongs to the same family as mangosteen (*Garcinia mangostana*), which is known locally in Sabah as “asam kandis” or cherry mangosteen. The present study was conducted to determine the phytochemicals content (total phenolic, flavonoid, anthocyanin, and carotenoid content) and antioxidant and acetylcholinesterase inhibition activity of the flesh and peel of *G. parvifolia*. All samples were freeze-dried and extracted using 80% methanol and distilled water. For the 80% methanol extract, the flesh of *G. parvifolia* displayed higher phenolic and flavonoid contents than the peel, with values of 7.2 ± 0.3 mg gallic acid equivalent (GAE)/g and 5.9 ± 0.1 mg rutin equivalent (RU)/g, respectively. Anthocyanins were detected in the peel part of *G. parvifolia* but absent in the flesh. The peel of *G. parvifolia* displayed higher total carotenoid content as compared to the flesh part with the values of 17.0 ± 0.3 and 3.0 ± 0.0 mg **β**-carotene equivalents (BC)/100 g, respectively. The free-radical scavenging, ferric reducing, and acetylcholinesterase inhibition effect of the flesh were higher as compared to the peel in both extracts. These findings suggested that the edible part of *G. parvifolia* fruit has a potential as a natural source of antioxidant and anti-Alzheimer's agents.

## 1. Introduction

In recent years, consumption of less-known fruits has become popular due to their health-promoting properties by virtue of their reported high phytochemicals content rather than because of taste or personal preference [1]. The prevention of several diseases has been proved to be highly related to the consumption of antioxidant-rich crops [2]. Antioxidants which exist naturally in food offer protection against some pathogenic events involving free radicals production and are associated with the prevention of many degenerative diseases such as cancer, aging, and atherosclerosis [3].

Sabah (situated in Borneo Island) is an administrative state of Malaysia which has about 200 species of indigenous edible fruits. Many of the fruits might be of benefit to human health but have not been commercialized. Most of the fruits grow naturally in the natural forest or jungle environment. *Garcinia parvifolia, *also known as “asam kandis” or “takob akob” among Sabahan, or “asam aur-aur” for Bruneian people, is one of the indigenous fruits of Borneo; in Sarawak, *G. parvifolia* is also known as “asam kundong”. This cherry-like fruit weighs about 11–19 g, of which the flesh constitutes almost 63% of its total weight, the remainder contributed by the peel. The diameter of the fruit is about 9–12 cm. The flesh of the fruit is white in colour with small seed inside it. The flesh is juicy and has a sweet and sour mangosteen-like taste. The unripe fruit of *G. parvifolia* is yellow in colour and changes into red colour when ripe. 

*G. parvifolia* prefers a humid tropical environment to grow well. Nowadays, it is occasionally cultivated by the local people. In terms of health, *G. parvifolia* benefits human by its medicinal properties, and these ethnomedicinal properties are used by people in Riau Province, Sumatra [4]. In addition to that, rubraxanthone isolated from *G. parvifolia* has been shown to display an inhibitory effect on platelet-activating factor receptor binding [5] and as such has potential in treatment of a variety of diseases such as bronchoconstriction-induced asthma, hyperacute organ-transplant rejection, inflammation, allergic reaction, thrombosis, endotoxin shock, cardiac anaphylaxis, and gastrointestinal ulceration. In light of these properties of *G. parvifolia*, this study was conducted to determine the phytochemicals content in different parts of the fruit and investigate the antioxidant and anticholinesterase potential using *in vitro* assays. 

## 2. Materials and Methods

### 2.1. Plant Materials and Sample Preparation

The fruit of *G. parvifolia* was collected from Sabah, Malaysia, during October to December 2011. The herbarium specimens were identified and deposited in BORNEENSIS, Universiti Malaysia Sabah, Malaysia. The fruits were cleaned, weighed, and separated into flesh and peel. Small cut pieces were freeze-dried and ground into fine powder using a dry grinder. The ground samples were sieved to get uniform size, then kept in an air-tight container, and stored in a freezer (−20°C) until further analysis. 

### 2.2. Extraction

Samples (0.1 g) were extracted separately for 2 hours with 80% methanol and with distilled water (each 2 mL, at a ratio of 1 : 20) at room temperature on an orbital shaker set at 200 rpm, as adapted from a previous method [6]. The mixture was then centrifuged at 1400 ×g for 20 min and the supernatant decanted into a 15 mL vial. The pellet was reextracted under identical conditions. Supernatant was combined and used for experiments on determination of phytochemicals content, antioxidant activity, and acetylcholinesterase inhibition.

### 2.3. Determination of Total Phenolic Content

Total phenolic content was determined using Folin-Ciocalteu reagent as adapted from a previous method [6] with slight modifications. Extract (300 *μ*L) was mixed with 2.25 mL of Folin-Ciocalteu reagent (previously diluted 10-fold with distilled water) and allowed to stand at room temperature for 5 min. Then we added 2.25 mL of sodium carbonate (60 g/L) solution to this. After 90 min at room temperature, absorbance was measured at 725 nm using a spectrophotometer. Standards of gallic acid in the concentration range 0 to 100 *μ*g/mL were run with the test samples, from which a standard curve was plotted. Result was expressed as mg gallic acid equivalents in 1 g of dried sample (mg GAE/g).

### 2.4. Determination of Total Flavonoid Content

Total flavonoid content was determined by a colorimetric method described previously [7] with slight modification. Briefly, 0.5 mL of extract was mixed with 2.25 mL of distilled water in a test tube followed by addition of 0.15 mL of 5% NaNO_2_ solution. We added 0.3 mL of a 10% AlCl_3_ *·*6H_2_O solution to this after 6 min, and allowed it to stand for another 5 min before 1.0 mL of 1 M NaOH was added and allowed to stand for another 5 min. The mixture was vortex-mixed, and the absorbance measured immediately at 510 nm using a spectrophotometer. Standards of rutin in the concentration range 0–100 *μ*g/mL were run with the test samples, from which a standard curve was plotted. Results were expressed as mg rutin equivalents in 1 g of dried sample (mg RE/g).

### 2.5. Determination of Total Anthocyanin Content

Total anthocyanin content was measured using a spectrophotometric pH differential protocol described by a previous method [8] with slight modification. Briefly, 0.5 mL of the extract was mixed thoroughly with 3.5 mL 0.025 M potassium chloride buffer pH 1. The mixture was mixed with vortex and allowed to stand for 15 min. The absorbance was then measured at 515 and 700 nm against a distilled water blank. The extract was then combined similarly with 0.025 M sodium acetate buffer pH 4.5 and the absorbance was measured at the same wavelengths after being allowed to stand for 15 min. The total anthocyanin content was calculated using the following equation:

(1) Total  anthocyanin  content  (mg/100 g  of  dried  sample)  =A×MW×DF×1000(ε×C),

where *A* is absorbance = (*A*_515_ − *A*_700_)  pH  1.0 − (*A*_515_ − *A*_700_)  pH  4.5, MW is molecular weight for cyanidin-3-glucoside (449.2), DF is the dilution factor of the samples, *ε* is the molar absorbtivity of cyaniding-3-glucoside (26900), and *C* is the concentration of the buffer in mg/mL. Results were expressed as mg of cyanidin-3-glucoside equivalents in 100 g of dried sample (mg C-3-GE/100 g dried sample).

### 2.6. Determination of Total Carotenoid Content

Total carotenoid content was measured by using a previous method [9] with slight modification. Sample (300 *μ*L) was added to 300 *μ*L distilled water and 600 *μ*L solvent (80% methanol or distilled water as appropriate) and the mixture mixed with 1.2 mL n-hexane. The mixture was centrifuged for 5 minutes at 4°C and absorbance of the hexane layer measured at 350 nm spectrophotometrically. Results were expressed as mg of *β*-carotene in 100 g of dried sample (mg BC/100 g dried sample).

### 2.7. DPPH Free-Radical Scavenging Assay

The scavenging activity of the extract was measured by using 1,1-diphenyl-2-picrylhydrazyl (DPPH) as a free-radical model and a method adapted from Magalhães et al. [10]. Aliquots (300 *μ*L) of sample, diluted in the concentration range 20–100 *μ*g/mL, or control (80% methanol or distilled water) were mixed with 3.0 mL of 500 *μ*M DPPH in absolute ethanol. The mixture was shaken vigorously and allowed to stand at room temperature for 30 min in the dark. Absorbance of the mixture was measured spectrophotometrically at 517 nm, and the free-radical scavenging activity was calculated as follows:

(2) Scavenging  effect (%)  =[1−{absorbance  of  sampleabsorbance  of  control}]×100.

The scavenging percentage of all samples was plotted. The final result was expressed as an EC_50_ value (the concentration of sample producing 50% scavenging of the DPPH radical; *μ*g/mL). Ascorbic acid in the same concentration range was used as a positive control.

### 2.8. Ferric Reducing/Antioxidant Power (FRAP) Assay

This procedure was conducted according to a previous method [11] with slight modification. The working FRAP reagent was produced by mixing 300 mM acetate buffer (pH 3.6), 10 mM 2,4,6-tripyridyl-s-triazine (TPTZ), solution and 20 mM FeCl_3_ *·*6H_2_O in a 10 : 1 : 1 ratio prior to use and heating them to 37°C in a water bath. A total of 3.0 mL FRAP reagent was added to a test tube and a blank reading was taken at 593 nm using spectrophotometer. A total of 100 *μ*L of selected plant extracts and 300 *μ*L of distilled water were added to the cuvette. A second reading at 593 nm was performed after 90 min of incubation at 37°C in water bath. The changes in absorbance after 90 min from initial blank reading were compared with standard curve. Standard of known Fe(II) concentrations were run using several concentrations between 0 and 1000 *μ*g/mL. A standard curve was then plotted. The final result was expressed as the concentration of antioxidant having a ferric reducing ability in 1 gram of sample (*μ*M/g).

### 2.9. ABTS Decolorization Assay

The ABTS decolorization assay was carried out according to the method described by Re et al. [12], with slight modification. Working ABTS solution (7 mM) and potassium persulfate (2.45 mM) were added into a beaker, and the mixture was allowed to stand 15 hours in the dark to generate an ABTS free-radical cation solution. The mixture was diluted with 80% methanol or distilled water in order to obtain absorbance of 0.7 ± 0.2 units at 734 nm. 200 *μ*L of methanolic or distilled water test solution was added to 2 mL of this working ABTS free-radical cation solution, the mixture vortexed for 45 seconds, and the resulting absorbance value read at 734 nm using microtiter plate reader. Standards of ascorbic acid in the concentration range 0–100 *μ*g/mL were run with the test samples, from which a standard curve was plotted. The final result was expressed as mg ascorbic acid equivalent antioxidant capacity in 1 g of sample (mg AEAC/g).

### 2.10. Acetylcholinesterase Inhibition Assay

The acetylcholinesterase inhibition assay was performed according to a previous method [13]. Phosphate buffer, 200 mM pH 7.7 (250 *μ*L), containing the fruit extract sample in the concentration range 50–250 *μ*g material/mL was mixed with DTNB solution (3.96 mg of DTNB and 1.5 mg sodium bicarbonate dissolved in 10 mL phosphate buffer pH 7.7; 80 *μ*L), and acetylcholinesterase (2 U/mL; 10 *μ*L) was added to the mixture. The mixture was incubated for 5 minutes at 25°C. Substrate (15 *μ*L of a solution of 10.85 mg acetylthiocholine iodide in 5 mL phosphate buffer) was added and the whole mixture incubated for a further 5 minutes. The colour developed in this time was measured by using microwell plate reader at 412 nm. The percent of inhibition was calculated by using the formula below.

(3) %  inhibition   =control  absorbance−sample/test  absorbancecontrol  absorbance  ×100%.

Galanthamine in the concentration range 0–100 *μ*g/mL was used as a positive control.

### 2.11. Statistical Analysis

All experiments were carried out in 3 replicates in 3 independent experiments. The results were presented as mean ± standard deviation (SD) using Prism 5 software. The data were statistically analysed by one-way ANOVA and Duncan posthoc test. The level of statistical significance was set at *P* ≤ 0.05. Pearson's correlation analysis was performed to correlate the phytochemicals and antioxidant and acetylcholinesterase inhibition activity in the samples. 

## 3. Results and Discussion

### 3.1. Total Phenolic Content

The result of this study showed that the total phenolic content was relatively higher in the flesh as compared to the peel in both the 80% methanol and the aqueous extracts (Table 1). However, there was no significant difference in the total phenolic content in the flesh and peel of *G. parvifolia* (*P* > 0.05) as assessed in the aqueous extract. Alcoholic solvents such as methanol and ethanol are widely used to extract polyphenols from natural sources. The present study showed that among the two extracts, the 80% methanol extract showed higher phenolic content than the aqueous extract. According to Adil et al. [14], the addition of small amount of organic solvents to an aqueous medium creates a more polar medium which facilitates extraction of phenolic compounds, such that mixtures of alcohols and water proved to be more efficient in extracting phenolic compounds compared to a monocomponent solvent. 

The total phenolic content of the flesh and peel of *G. parvifolia* is higher than that of the fruits of *G. atroviridis* and *G. prainiana* [15]. A study on *G. mangostana *showed that 92.6% of total phenolic acid present in aril and 97.3% of total phenolic acids in the peel was contributed by hydroxybenzoic acid derivative constitutents [16]. It is also suspected that the phenolic content in *G. parvifolia* has the same compound as *G. mangostana* since they belong to the same family. 

### 3.2. Total Flavonoid Content

Total flavonoid content in the flesh and the peel of *G. parvifolia* displayed the same trend as with the total phenolic contents in both aqueous and 80% methanolic extracts (Table 1). The highest total flavonoid content was shown in the 80% methanolic extract of the flesh of *G. parvifolia* with the value of 5.9 ± 0.1 mg RE/g, followed by the peel (80% methanol), flesh (aqueous extract) and peel (aqueous extract), with the values of 3.6 ± 0.3, 2.2 ± 0.1, and 1.2 ± 0.0 RE/g, respectively. The presence of flavonoids in the fruit flesh was also reported in a previous study on *Garcinia diocia *Blume [17].

### 3.3. Total Anthocyanin Content

The present study indicated that anthocyanins were present only in the peel of the fruit of *G. parvifolia* (Table 1) in both aqueous and 80% methanolic extract. The 80% methanol extract contained higher total anthocyanins content as compared to aqueous extract with the values of 4.4 ± 0.2 and 3.2 ± 0.1 mg C-3-GE/100 g dried sample (*P* < 0.05). The results were in agreement with the earlier literature; a study on *G. mangostana* showed no anthocyanin content in the flesh part of the sample but was present in the outer and inner pericarp [18]. 

### 3.4. Total Carotenoid Content

The peel of *G. parvifolia* had much higher total carotenoid contents as compared to the flesh part in both extracts tested (Table 1). The higher carotenoid content in the peel of *G. parvifolia* might contribute to the pink and red colour of the peel of this fruit. The carotenoid astaxanthin, that is mostly distributed in the *Garcinia *family [19], is suspected as being the major carotenoid compound in the peel of *G. parvifolia.*

### 3.5. DPPH Free-Radical Scavenging Activity

**Figure 1:**
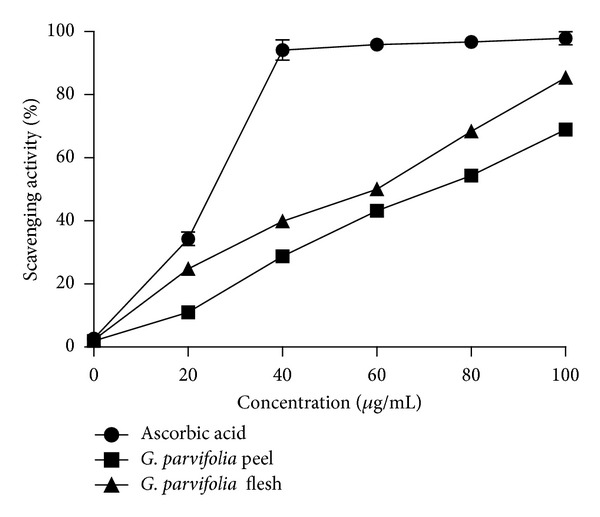
The scavenging activity of 80% methanol extract of different parts of the fruit of *G. parvifolia* assayed by DPPH free-radical scavenging method (*n* = 3).

**Figure 2:**
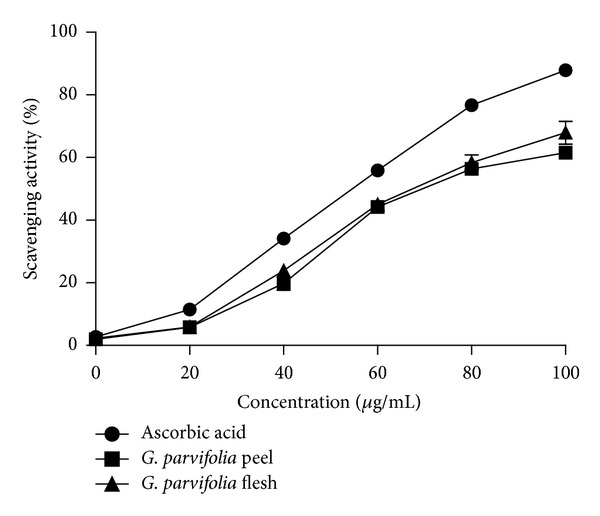
The scavenging activity of aqueous extract of different parts of the fruit of *G. parvifolia* assayed by DPPH free-radical scavenging method (*n* = 3).

The results for the 80% methanolic extracts showed that the flesh displayed slightly higher scavenging effects as compared to the peel (Figure 1). The scavenging activity of the aqueous extract displayed the same trend with the 80% methanolic extract (Figure 2). The flesh and peel of *G. parvifolia* showed significant difference in 80% methanol extract (*P* < 0.05) while no significant difference could be detected in the aqueous extract for scavenging free-radical activity (*P* > 0.05).

The EC_50_ value was determined to better quantify the radical scavenging activity in the samples (Table 2); the lower EC_50_ values indicate the stronger antioxidant potential. For 80% methanolic extract, the flesh showed the lower EC_50_ of the two fruit parts, with values of 58.0 ± 2.0 *μ*g/mL and 72.7 ± 2.5 *μ*g/mL. The same trend was also observed in the aqueous extract with EC_50_ values of 62.7 ± 3.1 *μ*g/mL and 76.7 ± 1.2 *μ*g/mL for the flesh and peel, respectively. 

Cheung et al. [20] reported that the scavenging activity of aqueous extracts was significantly lower than that of methanolic extracts of mushroom (*V. volvaco*) which is in agreement with this study that found that 80% methanol extract showed higher scavenging activity than the corresponding aqueous extracts (Figures 1 and 2).

The flesh of *G. parvifolia* displayed higher antioxidant properties than the peel of the fruit,which is similar to results for *Emblica officinalis* which displayed higher antioxidant activity in the edible part (flesh) as compared to the nonedible part (seed) [21]. 

### 3.6. Ferric Reduction Based on FRAP Assay

The reducing ability of the 80% methanol extracts of *G. parvifolia *was numerically higher in flesh followed by the peel. The same trend was also observed in the aqueous extract. However, there was no significant difference between the flesh and the peel of *G. parvifolia* in ferric reducing activity (*P* > 0.05; Table 2). The fact that the edible parts of fruits have more antioxidant properties as compared to the nonedible parts might be because of the higher phenolic and hydrolysable tannins content in the fruits [22]. 

### 3.7. ABTS Scavenging Assay

For the 80% methanol extracts, the higher scavenging activity was found in the flesh compared to the peel (Table 2). The value for the flesh of *G. parvifolia *was higher than that reported for the fruit of *G. atroviridis* but similar to that of *G. prainiana* [15]. Astaxanthin and zeaxanthin, two carotenoids that scavenge free radicals especially in lipid-soluble environment [23], are mainly distributed in the *Garcinia *family, and they are suspected of contributing to the antioxidant properties in the samples under study in this investigation.

### 3.8. Acetylcholinesterase Inhibition Activity

**Figure 3:**
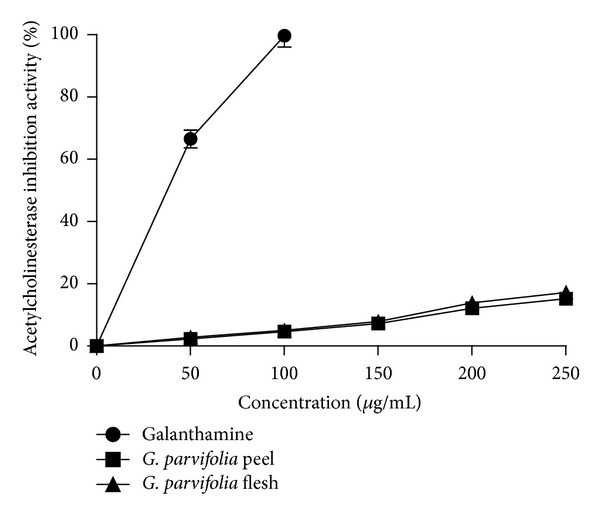
Acetylcholinesterase inhibition activity of 80% methanolic extract of different parts of the fruit of *G. parvifolia* (*n* = 3).

The 80% methanol extracts of the fruit parts of *G. parvifolia* inhibited acetylcholinesterase activity in a concentration-dependent manner in the range 50–250 *μ*g/mL (Figure 3), although the level of inhibition was small relative to that achieved by the positive control, galanthamine. The aqueous extracts of the fruit parts of G. parvifolia were essentially devoid of anticholinesterase inhibition activity (data not shown).

The inhibition towards acetylcholinesterase activity noted in this study is very similar to that of other *Garcinia *species such as *G. cambogia* which exhibit 14.3% inhibition activity towards acetylcholinesterase when tested at a concentration of 250 *μ*g/mL [24]. Screening of some Indian medicinal plants for acetylcholinesterase inhibitory activity also found that the methanolic extracts had greater effect as compared to aqueous extracts [25].

### 3.9. Relation between Phytochemicals Content, Antioxidant Activity, and Acetylcholinesterase Inhibition Effect

The phenolic compounds, flavonoids, anthocyanins, and carotenoids might be the phytochemicals that contribute to the antioxidant activity in the fruit extracts of *G. parvifolia*. Hence, correlation analysis was performed to investigate the relationship between the phytochemicals and antioxidant activity in the prepared extracts. From this analysis, the DPPH free-radical scavenging activity was positively correlated with the phenolic content of the extracts (*r* = 0.887). 

The results were in agreement with previous studies that showed that there was a strong correlation between the total phenolic content and antioxidant activity [26, 27]. Total flavonoid content in the sample was also positively highly correlated with antioxidant activity in the samples tested (*r* = 0.962), which is in agreement with an earlier study by Maisuthisakul et al. [28] that reported that antioxidant activity is closely related to the phenolic and flavonoid content. However, the antioxidant properties of the extracts were found to be moderately negatively correlated with the total anthocyanin content (*r* = −0.570) and total carotenoid content (*r* = −0.589).

Furthermore, the ferric reducing ability (FRAP) of the extracts was moderately correlated to the total phenolic (*r* = 0.832) and flavonoid content (*r* = 0.911), but moderately negatively correlated to the total anthocyanin content (*r* = −0.557) and total carotenoid content (*r* = −0.642) in the 80% methanol and aqueous extracts. This result was in agreement with a previous study [29] which showed that the phenolic and flavonoid content are highly related to the reducing ability of the samples. 

The acetylcholinesterase inhibition activity showed moderate positive correlation with the antioxidant activity as determined by the DPPH, FRAP, and ABTS assays (*r* = 0.769, 0.751, and 0.820, resp.). This observation of a positive correlation of anticholinesterase activity with antioxidant activity was supported by a previous study on *G. cambogia* extracts which also reported that the antioxidants activity in the samples contributed to the acetylcholinesterase inhibition activity [24].

## 4. Conclusions

In conclusion, extracts of the fruit of *G. parvifolia* demonstrated a potential as natural resource of antioxidants and acetylcholinesterase inhibitor agent with acceptable amount of phenolic and flavonoid content. Efforts on the promotion and utilization of this fruit should be done comprehensively for public health benefits as it possesses promising antioxidant and anticholinesterase properties, the latter being particularly promising for the treatment of Alzheimer's disease. 

## Figures and Tables

**Table 1:** Content of total phenolics, total flavonoids, total anthocyanins, and total carotenoids in extracts of the fruit of *G. parvifolia*.

Samples	Total phenolics^1^	Total flavonoids^2^	Total anthocyanins^3^	Total carotenoids^4^
80% methanol				
Flesh	7.2 ± 0.3^c^	5.9 ± 0.1^d^	ND	3.0 ± 0.0^b^
Peel	5.3 ± 0.1^b^	3.7 ± 0.3^c^	4.4 ± 0.2^b^	17.0 ± 0.3^a^
Aqueous				
Flesh	2.3 ± 0.1^a ^	2.2 ± 0.1^b^	ND	1.8 ± 0.8^b^
Peel	1.8 ± 0.1^a ^	1.2 ± 0.0^a^	3.2 ± 0.1^a^	15.9 ± 0.9^a^

Values are presented as mean ± SD (*n* = 3) which, with different letters (within column), are significantly different at *P* < 0.05. ND: not detected.

^1^Total phenolic content was expressed as mg gallic acid equivalents in 1 g of dried sample (mg GAE/g).

^2^Total flavonoid content was expressed as mg rutin equivalents in 1 g of dried sample (mg RE/g).

^3^Total anthocyanin content was expressed as mg of cyanidin-3-glucoside equivalents in 100 g of dried sample (mg C-3-GE/100 g dried sample).

^4^Total carotenoid content was expressed as mg of *β*-carotene equivalents in 100 g of dried sample (mg BC/100 g dried sample).

**Table 2:** Antioxidant properties of extracts of different parts of the fruit of *G. parvifolia*, assessed by three different assays.

Samples	DPPH assay (EC_50_ value)^1^	DPPH assay (%)^2^	FRAP assay^3^	ABTS assay^4^
80% methanol				
Flesh	58.0 ± 2.0^b^	85.4 ± 1.3^a^	16.6 ± 3.8^a^	32.7 ± 8.5^a^
Peel	72.7 ± 2.5^a^	68.9 ± 0.9^b^	14.8 ± 0.4^a^	22.6 ± 1.9^a,c^

Aqueous				
Flesh	62.7 ± 3.1^b^	67.9 ± 3.6^b^	14.8 ± 1.6^a^	20.0 ± 1.0^b,c^
Peel	76.7 ± 1.2^a^	61.5 ± 0.4^b^	12.0 ± 1.0^a ^	18.2 ± 0.1^b,c^

Values are presented as mean ± SD (*n* = 3) which, with different letters (within column), are significantly different at *P* < 0.05.

^1^DPPH free-radical scavenging activity was expressed as EC_50_ (*μ*g/mL).

^2^DPPH free-radical scavenging activity percentage in mg sample (%).

^3^FRAP was expressed as mM ferric reduction to ferrous in 1 g of dry sample.

^4^ABTS free-radical scavenging activity was expressed as mg ascorbic acid equivalent antioxidant capacity (AEAC) in 1 g of dry sample.
